# Intralaryngeal thyroglossal duct cyst

**DOI:** 10.11604/pamj.2015.22.294.8264

**Published:** 2015-11-24

**Authors:** Mohamed Mliha Touati, Haddou Ammar

**Affiliations:** 1 ENT Department, Military Hospital Avicenna, Marrakech, Morocco

**Keywords:** Thyroglossal duct cyst, laryngeal extention, embryogenesis

## Image in medicine

**Figure 1:**
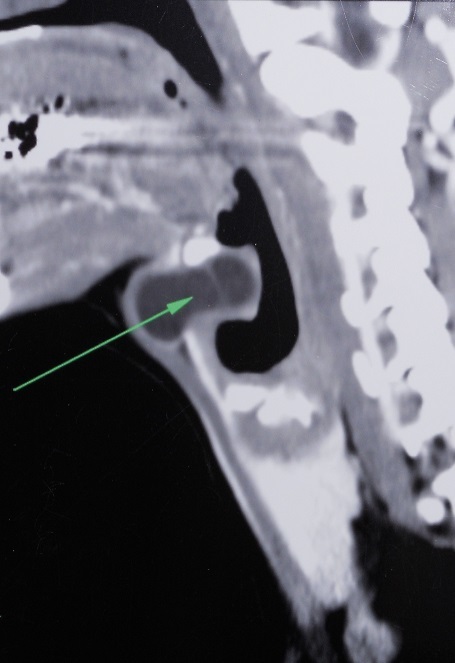
Cervical CT scann showing a bilobular cystic mass with intralryngeal extention

Thyroglossal duct cyst (TDC) is the most common congenital neck mass, approximately 7% of the Population has a TDC. It results from incomplete resorption of the thyroglossal duct during embryogenesis. A 31-year-old male patient presented with a painful left paramedian neck swelling for two months. Physical examination revealed a cystic mobile swelling; the lesion disappeared on hyperextension of the neck and reappeared on lateral rotation to the left side. Cervical CT scann revealed a bilobular cystic mass on the prominentia thyroidea, and extending deeply into the preepiglottic region, turning medially from the upper border of the thyroid cartilage (Figure 1). Surgery revealed a cystic lesion with intralaryngeal extension through a defect in the thyrohyoid membrane. In the last step of the surgery, as described in the Sistrunk procedure, the central portion of the hyoid bone was included in the specimen. The tract was followed to the tongue base and then cut after ligation. Histopathology confirmed the diagnosis of an inflamed TDC. During the 6-month postoperative follow-up of the patient, no finding consistent with recurrence was detected in the surgical site. TDC may be located at the level of the hyoid (15-50%), the suprahyoid (20-25%), or the infrahyoid (25-65%). The intralaryngeal location of a thyroglossal duct cyst is rare and only a few cases have been reported, which makes this case interesting. TDC can extend from the thyroid cartilage. It can push or destroy the thyrohyoid membrane and then progress to the laryngeal ventricle, which causes it to be confused with saccular cyst or laryngocele. Airway obstruction due to intralryngeal thyroglossal duct cyst can cause the disease to show a fatal course. Evaluation of laryngeal structures with preoperative endoscopic and radiological techniques is an important step that can help the diagnosis. Sistrunk procedure should be administered for such cases.

